# Thyroid Disruption by Di-n-Butyl Phthalate (DBP) and Mono-n-Butyl Phthalate (MBP) in *Xenopus laevis*

**DOI:** 10.1371/journal.pone.0019159

**Published:** 2011-04-22

**Authors:** Ouxi Shen, Wei Wu, Guizhen Du, Renping Liu, Lugang Yu, Hong Sun, Xiumei Han, Yi Jiang, Wei Shi, Wei Hu, Ling Song, Yankai Xia, Shoulin Wang, Xinru Wang

**Affiliations:** 1 The Center for Disease Control and Prevention of Suzhou Industrial Park, Suzhou, China; 2 Key Laboratory of Reproductive Medicine, Institute of Toxicology, School of Public Health, Nanjing Medical University, Nanjing, China; 3 Key Laboratory of Modern Toxicology (Nanjing Medical University), Ministry of Education, China; 4 Jiangsu Provincial Center for Disease Control and Prevention, Nanjing, China; 5 Department of Obstetrics and Gynecology, The First Affiliated Hospital of Nanjing Medical University, Nanjing, China; 6 State Key Laboratory of Pollution Control and Resource Reuse, School of the Environment, Nanjing University, Nanjing, China; Ecole Normale Supérieure de Lyon, France

## Abstract

**Background:**

Di-n-butyl phthalate (DBP), a chemical widely used in many consumer products, is estrogenic and capable of producing seriously reproductive and developmental effects in laboratory animals. However, recent *in vitro* studies have shown that DBP and mono-n-butyl phthalate (MBP), the major metabolite of DBP, possessed thyroid hormone receptor (TR) antagonist activity. It is therefore important to consider DBP and MBP that may interfere with thyroid hormone system.

**Methodology/Principal Findings:**

Nieuwkoop and Faber stage 51 Xenopus laevis were exposed to DBP and MBP (2, 10 or 15 mg/L) separately for 21 days. The two test chemicals decelerated spontaneous metamorphosis in *X. laevis* at concentrations of 10 and 15 mg/L. Moreover, MBP seemed to possess stronger activity. The effects of DBP and MBP on inducing changes of expression of selected thyroid hormone response genes: thyroid hormone receptor-beta (*TRβ*), retinoid X receptor gamma (*RXRγ*), alpha and beta subunits of thyroid-stimulating hormone (*TSHα* and *TSHβ*) were detected by qPCR at all concentrations of the compounds. Using mammalian two-hybrid assay *in vitro*, we found that DBP and MBP enhanced the interactions between co-repressor SMRT (silencing mediator for retinoid and thyroid hormone receptors) and TR in a dose-dependent manner, and MBP displayed more markedly. In addition, MBP at low concentrations (2 and 10 mg/L) caused aberrant methylation of *TRβ* in head tissue.

**Conclusions:**

The current findings highlight potential disruption of thyroid signalling by DBP and MBP and provide data for human risk assessment.

## Introduction

Di-n-butyl phthalate (DBP) are high production volume plasticizers present in food products such as butter and infant formula, as well as in a variety of cosmetics [1]–[3]. DBP can be detected in air, soil and aquatic ecosystems due to its continuous release into the environment. Given its ability to intercalate into the ecosystem, it is not surprising that DBP and its major metabolite, mono-n-butyl phthalate (MBP), have been identified in tissues from several human subpopulations [4]–[6]. Recent work from our laboratory showed that the reproductive system was damaged severely by DBP, resulting in the developmental condition of hypospadiac male offspring [7]. Additionally, using a recombinant CV-1 cell line containing a L-3,5,3′-triiodothyronine (T_3_)-dependent reporter gene, UAS-tk-Luc which was cotransfected with Gal4-L-TR into CV-1 cell line, we found that DBP and MBP possessed TR antagonist activity [8]. Furthermore, comparing to DBP, the metabolite MBP has been proved to be a more potent antagonist [8].

The effects of endocrine-disrupting chemicals (EDCs) on the sex steroid system have attracted the most attention over the past decades, with reports that environmental contaminants act either as estrogenic, androgenic, anti-estrogenic or anti-androgenic agents and consequently affect the regulation and function of the reproductive system and sexual development [9], [10]. Thyroid hormones regulate a variety of biological processes associated with development, somatic growth, metabolism, energy provision, and reproduction in vertebrates and thus, effects of EDCs on the thyroid system may pose a hazard to human and wildlife health [11]–[13]. Indeed, it has been noted that functions of the thyroid system are susceptible to disruption by EDCs [14], [15].

The metamorphosis of *Xenopus laevis* (*X. laevis*) is regulated by the hypothalamus-pituitary-thyroid axis [16]. The synthesis and secretion of the thyroid hormone are stimulated by the pituitary thyroid-stimulating hormone (TSH) [17]. In peripheral tissues thyroid hormone effects are mediated by binding to specific nuclear thyroid receptors, TRα and TRβ. During metamorphosis the expression of TRα is constitutive, whereas expression of TRβ is more variable and closely correlated with changes in circulating thyroid hormone [18], [19]. Metamorphic development of *X. laevis* is a thyroid hormone-dependent process that can be inhibited by exposing to chemicals which inhibit its synthesis [20]. Based on this knowledge, metamorphic development of *X. laevis* has been proposed as a unique biological system to specifically investigate EDC effects on thyroid hormone action [21], [22]. Studies of Lee et al. [23] indicated that DBP significantly affected development of *X. laevis* embryos at low, environmentally relevant concentrations. However, there have been few reports concerning the effects of DBP and MBP on metamorphosis of *X. laevis* and the molecular mechanisms of thyroid system disruption.

In the present study, we investigated various morphological and molecular endpoints to detect thyroid system disruption in *X. laevis* tadpoles by the compounds DBP and MBP. We showed that metamorphic retardation caused by DBP and MBP in a concentration-dependent manner. Additionally, the potential impact on thyroid system was investigated by assessing mRNA levels of *TRβ*, *RXRγ* (retinoid X receptor gamma), *TSHα* and *TSHβ*, which are sensitive molecular endpoints for anti-thyroidal EDCs. The effects of DBP and MBP on the interaction between SMRT (silencing mediator for retinoicd and thyroid hormone receptors) and TR were examined using a mammalian two-hybrid assay. We also investigated aberrant methylation of the *TRβ* gene that could act as an alternative mechanism in disrupting the function of the *TRβ* gene in *X. laevis* treated with chemicals.

## Materials and Methods

### Ethics Statement

All animal experiments were carried out in compliance with, and approved by, the Institutional Animal Care and Use Committee.

### Experimental Animal

Spawning of adult *X. laevis* (Nasco, USA) was induced in accordance with the method of Kloas et al. [24]. Tadpoles were maintained in a 20-L glass aquarium containing dechlorinated water at 22°C±1°C, pH 7.0±0.5, with a photoperiod of 12 h light and 12 h dark during all phases of the experiments described below. Tadpoles were fed with Nasco Frog Brittle (Nasco, USA) throughout the pre-exposure period (5 days after fertilization) and during the entire test period. Developmental stages of tadpoles were determined according to the Normal Table of *X. laevis* [25].

### Exposure System

**Figure 1:**
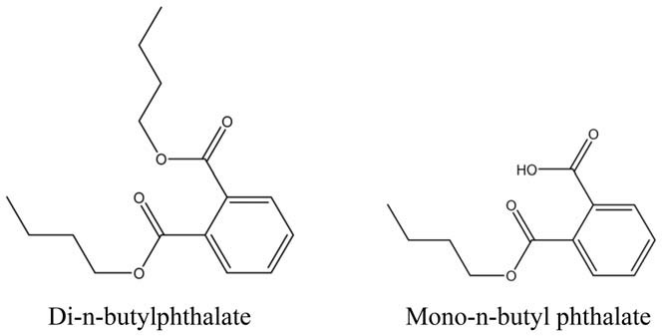
Chemical structures of DBP and MBP.

The source, purity, CAS and abbreviation of chemicals are listed in Table 1. L-3,5,3′-triiodothyronine (T_3_; ≥98%) were purchased from Sigma Chemical Co. (St. Louis, MO, USA). The structures of chemicals in this study are indicated in Figure 1. DBP and MBP (both from Tokyo Kasei, Japan) were dissolved in dimethyl sulfoxide (DMSO; Sigma, USA) at 250 mg/L and 50 mg/L as stock solutions and diluted in dechlorinated water at appropriate concentrations. The stock solutions of DBP and MBP were stored at −20°C in the dark until they are ready to use. T_3_ was first dissolved in DMSO at 10^−1^ M as stock solutions and then diluted in DMSO at 10^−3^ M before diluted in dechlorinated water at 1 nM (6.51×10^−4^ mg/L). Groups of twenty tadpoles reached stage 51 (14–16 days post-fertilization) were placed in 5 L of dechlorinated water containing concentrations of 2, 10, 15 mg/L DBP and MBP, 1 nM T_3_ and 0.005% DMSO as solvent control for 21 days. Test solutions were replaced every 2 days. The tadpoles were daily monitored for their developmental stages. On day 22, developmental stage, whole body length, interocular distance and body-to-tail length ratio were determined in animals in all aquariums. Subsets of three tadpoles per aquarium with average stage were randomly selected to collect head tissue for RNA extraction and six tadpoles for DNA extraction. The remaining tadpoles were still kept in aquariums until they reached stage 57.

**Table 1:** Data on test chemicals.

Chemicals	Abbreviation	Supplier	CAS no.	Purity (%)
Di-n-butylphthalate	DBP	Tokyo Kasei	84-74-2	>99
Mono-n-butyl phthalate	MBP	Tokyo Kasei	131-70-4	>99

Tokyo Kasei: Tokyo Kasei Kogyo Co., Ltd.

### Quantitative Real-time Polymerase Chain Reaction

Total RNA was extracted using TRIzol reagent as described by the manufacturer (Invitrogen Life Technologies Co, USA). The quantity of specific RNA for target genes in each sample was estimated by quantitative real-time polymerase chain reaction (qPCR) in a volume of 10 µL with 384-well plates using ABI Prism 7900HT (Applied Biosystems, Foster City, CA, USA). Each PCR was run in triplicate to control for PCR variation. The thermocycler program included a step of denaturation at 95°C (10 min), and 40 cycles of 95°C (15 sec) and 60°C (1 min). Each 10-µL DNA amplification reaction containing 0.5 µL of diluted cDNA, 0.5 µM each of forward and reverse primer, 200 µM dNTPs, 1× PCR buffer, 1.25 U of Ex *Taq* Hot Start DNA Polymerase (Takara Bio, Tokyo, Japan) and 0.2 µmol of EvaGreen (Biotum, USA). We included controls lacking cDNA template or *Taq* DNA polymerase to determine the specificity of target cDNA amplification. Triplicate data obtained from the amplification of each target cDNA were averaged and normalized to *gapdh* (GenBank Accession Number NM_001087098). See Table S1 for primer sequences.

### Mammalian Two-hybrid Assay

#### Plasmid Constructs

The plasmid pCMX-VP-F-SMRT contains the full length of SMRT was provided by Professor Ronald M. Evans (Gene Expression Laboratory, Howard Hughes Medical Institute, San Diego, CA, USA). The C-terminal domain of SMRT (acids 1197–1495) which is involved in receptor interaction [26] was amplified by the following primer pairs: forward: 5′-ATTCGAATTCGAGGACGGTATTGAACCTGTGTCCC-3′, reverse: 5′-ATTCGGATCCTCACTCGCTGTCGGAGAGTGTCTCG-3′. The fragment of the C-terminal domain of SMRT subsequent restriction enzyme digested with EcoRI and BamHI was inserted between EcoRI and BamHI site of pM which was a Gal4 DNA-binding domain cloning vector (Clontech, USA) to create the new plasmid pM-SMRT. The fragment of ligand binding domain (LBD) of hTRβ obtained from the plasmid pCMX-Gal-L- hTRβ (from professor Ronald M. Evans) using restriction enzyme EcoRI and BamHI and plasmid pVP16 (Clontech, USA) digested with EcoRI and BamHI were ligated to create the new plasmid pVP16-L- hTRβ.

#### Transient Expression Assay

Green monkey kidney fibroblast (CV-1) cell line (Chinese Cell Center, Beijing) was maintained in Dulbecco's modified Eagle's medium (DEME) (Sigma) supplemented with 10% fetal bovine serum (FBS; Invitrogen Life Technologies Co, USA), 100 U/mL penicillin (Sigma) and 100 µg/mL streptomycin (Sigma) at 37°C in an atmosphere containing 5% CO_2_. The host cells were plated in 48-well microplate in the phenol red-free DMEM medium containing 10% charcoal-dextran-stripped FBS (CDS-FBS). Twelve hours later the CV-1 cells were transfected with 100 ng pM-SMRT, 200 ng pVP16-L- hTRβ and 200 ng Gal4 responsive luciferase reporter pUAS-tk-luc using 2.5 µg Sofast™ (Sunma Company, Xiamen, China) transfection reagent per well. After an incubation period of 12 h, cells were treated with the test solutions. Exposure was carried out over 24 h to 1 nM T_3_, solvent-controls and test compounds (1.0×10^−9^–1.0×10^−5^ M in 10-fold dilution steps). Then cells were harvested for measurements of Luc activity according to the manufacturer's instructions (Luciferase Reporter Assay system kit, Promega, Madison, WI, USA). The relative transcriptional activity was converted to fold induction above the corresponding vehicle control value (n-fold).

### Bisulfite Sequencing

Bisulfite sequencing was used to characterize the DNA methylation patterns and changes at the promoter region of *TRβ* gene in *X. laevis*. Three samples of head were mix together as a pool and each treatment group contain two pools. Genomic DNA was isolated from head pools of different groups of *X. laevis*. Extracted DNA was treated and modified with a sodium bisulfite procedure using the EpiTect Bisulfite Kit (Qiagen), according to manufacturer's instructions. The amplified region included 18 CpG sites (CpGs) and spanned a 267 bp sequence (GenBank Accession Number Z30971; nucleotides 591–855). The bisulfite primers for *TRβ* gene were designed using the MethPrimer (www.urogene.org/methprimer) system [27] and listed in Table S1. Each DNA was amplified by PCR as follows: a PCR reaction mix containing 5 µL of the bisulfite-treated DNA, 0.5 µM each of forward and reverse primer, 200 µM dNTPs, 1× PCR buffer, 1.25 U of Ex *Taq* Hot Start DNA Polymerase (Takara Bio, Tokyo, Japan) in a total volume of 25 µL. After activation of the polymerase at 95°C for 10 min, DNA was amplified in 35 cycles for 30 s at 95°C, 40 s at 53°C and 1 min at 72°C followed by a final extension at 72°C for 10 min. Amplified products were purified with the QIAquick Gel Extraction Kit (Qiagen) and cloned into the pCR2.1 vector (Invitrogen Life Technologies Co, USA), and individual clones were sequenced using M13 reverse primer and an automated ABI Prism 3730xl Genetic Analyser (Applied Biosystems, Foster city, CA). Approximately 15 different clones from each PCR product were sequenced to characterize the methylation status of the CpGs. The methylation status of all CpGs present in the sequences was analyzed using the BiQ Analyzer software [28].

### Statistical Analysis

The data were presented as mean ± standard deviation (SD). Statistic analyses for morphological parameters, gene expression and mammalian two-hybrid assay were performed by one-way analysis of variance (ANOVA), followed by Duncan's multiple comparison test. The chi-squared test was used to evaluate the differences of complete methylation of *TRβ* between different treatment groups and control. All statistical analyses were carried out using Stata (Version 9.0, StataCorp, LP), and *P*≤0.05 were considered to be significant.

## Results

### Inhibition of Metamorphosis by DBP and MBP

Exposure to both DBP and MBP resulted in a concentration-dependent inhibition of metamorphic development. The median stage of tadpoles immersed in 15 mg/L DBP and MBP was stage 54 and 53 while tadpoles kept in the solvent control and positive control reached stage 57 and 58 respectively (Table 2). Whole body length, interocular distance and body-to-tail length ratio that were measured to assess the growth of tadpoles are depicted in Table 2. The test chemicals did not affect the body-to-tail length ratio of the tadpoles, but significantly reduced the interocular distance at the highest concentration. Further, the whole body length of tadpoles treated with 10 and 15 mg/L MBP was significantly less than control while only 15 mg/L DBP was.

**Table 2:** Summary of morphological parameters in xenopus laevis of 21-Day exposure.

Endpoint	DMSO	T_3_ (mol/L)	DBP (mg/L)			MBP (mg/L)		
	0.005%	10^−9^	2	10	15	2	10	15
Developmental stage	57 (56–57)^a^	58 (56–59)*	57 (55–57)	56 (53–57)*	54 (53–54)*	57 (55–57)	55 (54–57)*	53 (53–55)*
Whole body length (mm)	53.8±3.3^b^	47.7±5.4	51.7±9.4	44.8±5.5	38±1.9*	52.1±4.9	42.4±2.2*	37.8±4.2*
Interocular distance (mm)	8.9±0.4^b^	7.1±1.5	8.7±1.2	7.7±1.1	6.7±0.5*	8.8±0.8	8.2±0.5	7.0±0.6
Body-to-tail length ratio	2.2±0.2^b^	2.2±0.2	2.0±0.2	2.1±0.1	2.1±0.1	2.1±0.2	1.9±0.1	1.9±0.5

*Note.* Developmental stage, whole body length, interocular distance and body-to-tail length ration were determined for all tadpoles on exposure day 22.

a Values given as median stage with the total range of stages in parentheses.

b Values given as means and standard deviations.

*Significantly different from the DMSO control (*P*<0.05).

### Gene Expression Analysis

**Figure 2:**
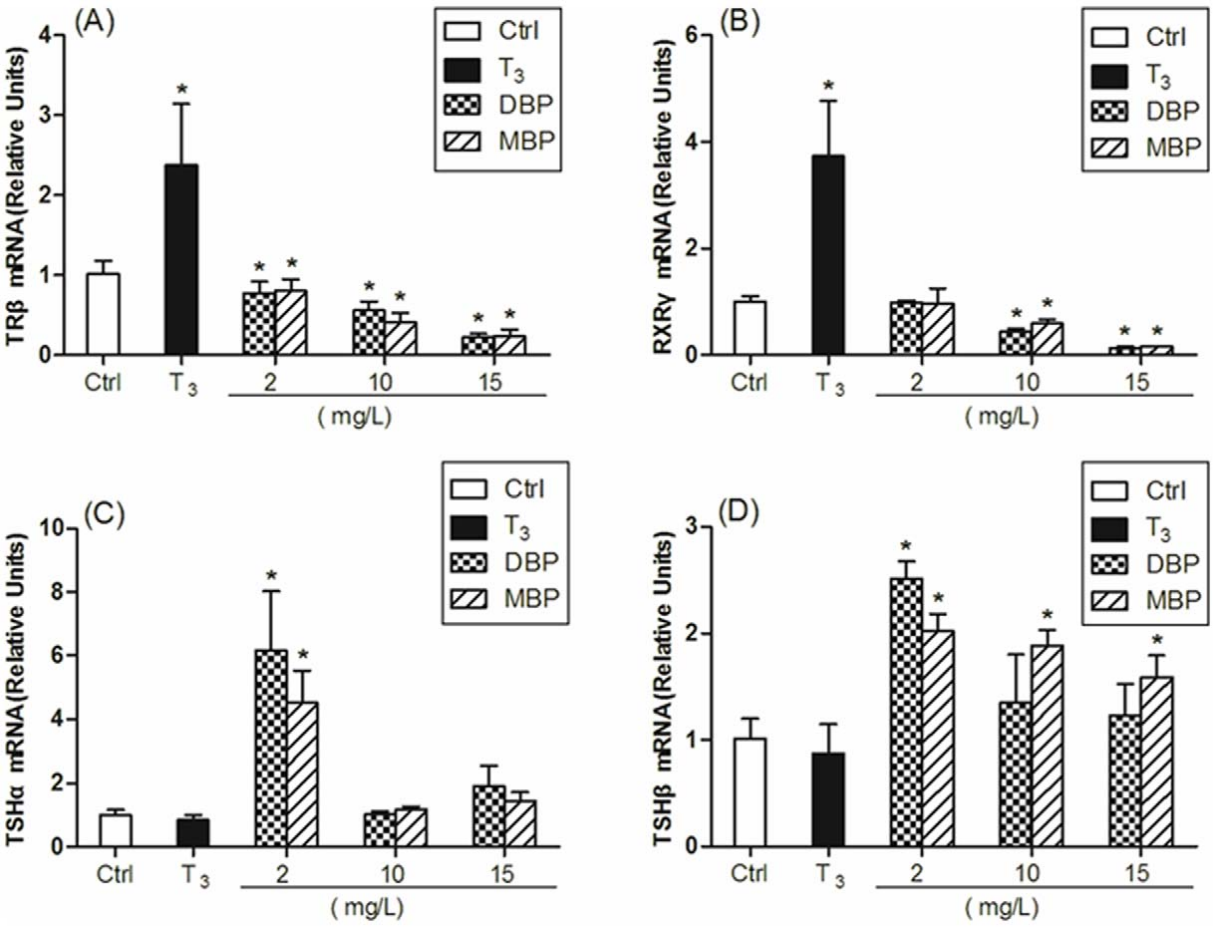
Effects of DBP and MBP exposure on mRNA expression of *TRβ* (panel A), *RXRγ* (panel B), *TSHα* (panel C) and *TSHβ* (panel D) in head of tadpoles exposed day 21. Treatment with 0.005% DMSO served as a solvent control. Exposure of tadpoles was initiated at stage 51, and head tissue was sampled when control tadpoles reached stage 57. *TRβ*, *RXRγ*, *TSHα* and *TSHβ* values were normalized by *gapdh* values, and results were expressed relative to the control. Data are shown as mean ± standard deviations (SD) (n = 3 tadpoles per treatment group). Asterisks denote significant differences from controls (*P*<0.05).

When determined in head tissues of tadpoles exposed day 21, the expression of *TRβ* and *RXRγ* were significantly increased in tadpoles exposed to T_3_, while no significant differences was detected in expression of *TSHα* and *TSHβ* between control and T_3_ treatment groups (Figure 2). The *TRβ* mRNA levels were down-regulated significantly in all the 2, 10 and 15 mg/L DBP/MBP treated groups compared with the control group (Figure 2A). The mRNA levels of *RXRγ* decreased significantly in the 10 and 15 mg/L DBP/MBP treated groups compared with the control group. However, no significant differences was observed between the lowest concentration of DBP/MBP groups (2 mg/L) and the control group (Figure 2B). With regard to the mRNA levels of *TSHα*, no significant differences was observed between the high concentrations (10 and 15 mg/L) of DBP/MBP groups and the control group (Figure 2C). At 10 and 15 mg/L exposure levels, the mRNA level of *TSHβ* increased significantly only in 10 and 15 mg/L MBP treated groups compared with the control group (Figure 2D). However, mRNA expression of both TSH subunits was dramatically increased in low concentration of DBP and MBP (2 mg/L; Figure 2C and D).

**Figure 3:**
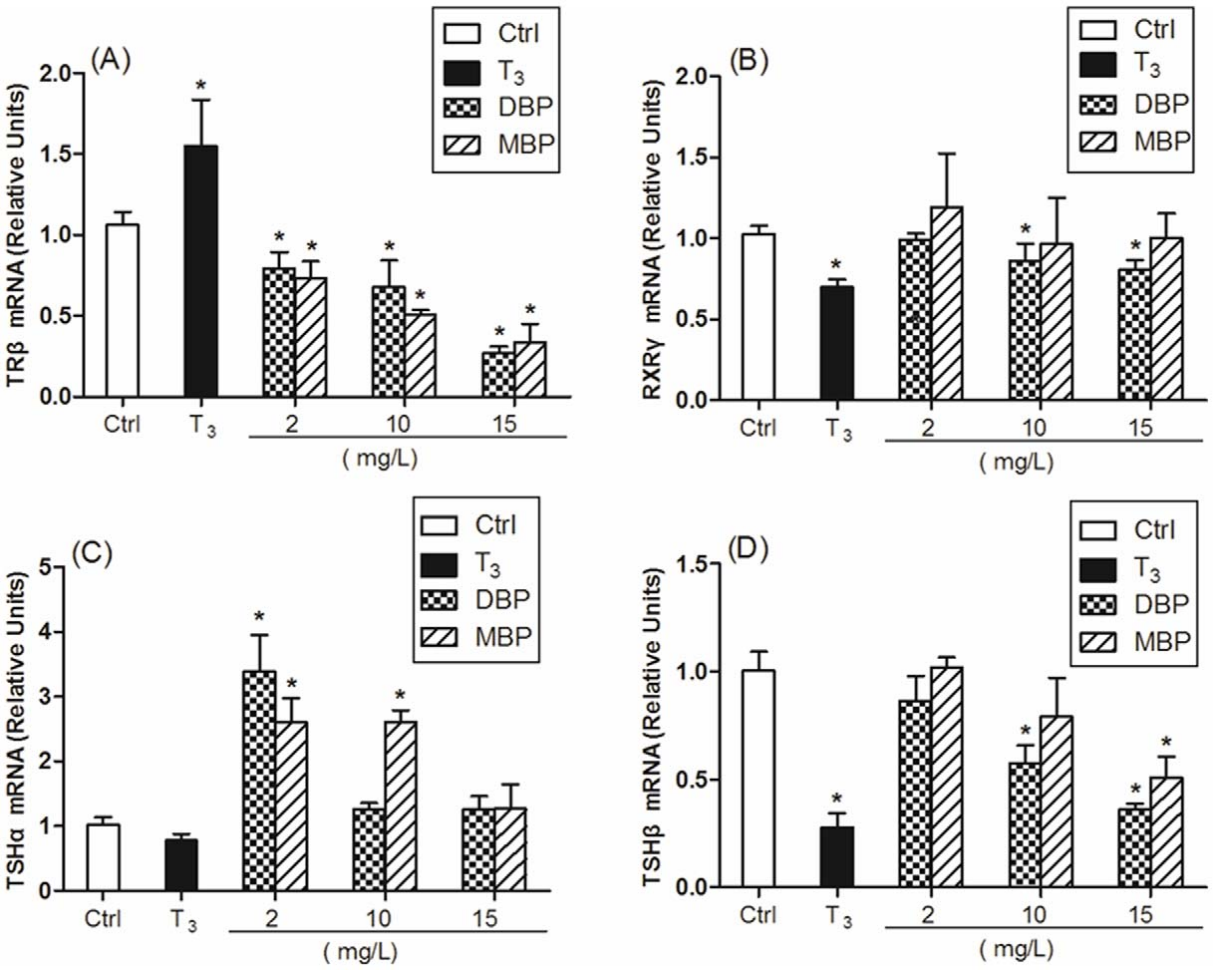
Effects of DBP and MBP exposure on mRNA expression of *TRβ* (panel A), *RXRγ* (panel B), *TSHα* (panel C) and *TSHβ* (panel D) in head tissue of tadpoles reached stage 57. Treatment with 0.005% DMSO served as a solvent control. *TRβ*, *RXRγ*, *TSHα* and *TSHβ* values were normalized by *gapdh* values, and results were expressed relative to the control. Data are shown as mean ± SD (n = 3 tadpoles per treatment group). Asterisks denote significant differences from controls (*P*<0.05).

Considering the different stages of tadpoles may be a factor that would influence the expression of the target genes, we also analyzed the expression of these genes of the tadpoles reached stage 57 in different groups. In head tissue, stage 57 tadpoles almost had the same tendency as *TRβ*, *RXRγ* and *TSH* subunits expression with the tadpoles at different stages except for down-regulation of *RXRγ* caused by T_3_ and down-regulation of *TSHβ* caused by DBP and MBP (Figure 3).

### DBP and MBP Recruit SMRT

**Figure 4:**
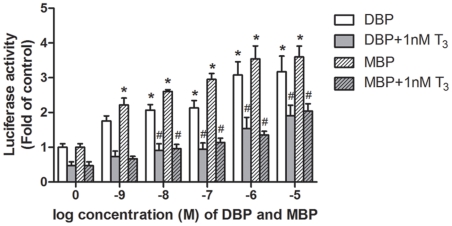
The effects of DBP and MBP on the interaction between TR and SMRT. Cells were treated with increasing concentrations of DBP (10^−5^ M = 2.78 mg/L) and MBP (10^−5^ M = 2.22 mg/L) alone or with 1 nM T_3_ (6.51×10^−4^ mg/L) respectively. Data are shown as mean ± SD of three independent experiments, and the activities are presented as fold of solvent control. *P<0.05 compared with solvent control. #*P*<0.05 compared with 1 nM T_3_.

The effects of DBP and MBP on the interaction between SMRT and TR were examined using a mammalian two-hybrid assay. The C-terminal domain of SMRT (nucleotides 1197–1495) which is involved in receptor interaction, was fused to the Gal4-DBD (DNA binding domain). The LBD of hTRβ was fused to the transcriptional activation domain of VP16 to detect the interaction between Gal4-SMRT and VP16-TR. DBP and MBP enhanced the interaction between SMRT and TR even though the addition of 1 nM T_3_ released the interaction (Figure 4). The addition of 1 nM T_3_ released totally the interaction with the 10^−9^ M dose but this reversal is not complete from 10^−8^ M until 10^−5^ M concentrations indicating persistency effects for both DBP and MBP at higher doses (Figure 4).

### Methylation Status of TRβ Gene

A total of 210 clones were studied for methylation status of *TRβ* gene in head tissue of *X. laevis* (see Figure S1). We here show that the promoter region of *TRβ* was almost fully methylated (96.8%, with CpG 11 unmethylated in one clone) in control group. Complete promoter methylation in the *TRβ* gene was identified in all groups (see Figure S1). The frequency of clones with complete methylation in all treated groups were lower than in control group, especially the groups treated with 2 and 10 mg/L MBP (*P*<0.05; Table 3).

**Table 3:** Methylation status of promoter region of *TRβ* gene in *X. laevis* head.

Methylation	Control	DBP (mg/L)			MBP (mg/L)		
		2	10	15	2	10	15
Completely methylated clone [n (%)]	30 (96.8)	27 (90)	27 (93.1)	25 (80.6)	19 (63.3)	21 (70.0)	24 (82.8)
Incompletely methylated clone [n (%)]	1 (3.2)	3 (10)	2 (6.9)	6 (19.4)	11 (36.7)*	9 (30.0)*	5 (17.2)

*P<0.05 when compared between treated and control groups.

## Discussion

Recent studies have shown that *TRβ*, *RXRγ*, *TSHα* and *TSHβ* mRNA expression analysis could serve as a sensitive molecular approach to study effects of environmental compounds on the thyroid system in *X. laevis* tadpoles [29], [30]. No significant changes in developmental stages of tadpoles were induced by low concentrations (2 mg/L) of DBP and MBP perhaps due to the capacity of the hypothalamus-pituitary-thyroid axis to maintain thyroid hormone homeostasis. Nonetheless, gene expression changes caused by DBP and MBP at doses ranging from 2 to 15 mg/L were observed. Previous study has suggested that BPA suppressed the expression of *TRβ* possibly by counteracting the endogeneous thyroid hormones [31]. Thus, we inferred that the down-regulated expression of *TRβ* caused by DBP and MBP may also go through the mechanism. It has been demonstrated that expression of *TRβ* is altered along with the stage of tadpoles. We analyzed the expression of *TRβ* in head tissue of tadpoles at stage 57 from all groups, and the down-regulation of *TRβ* expression in treatment groups of DBP and MBP were also detected.

It is generally accepted that TR as heterodimer with RXRs binding to the thyroid response element (TRE) to regulate the transcription of target genes [32], [33]. *RXRr* interacts more strongly with *TRβ* than other isoforms [34]. The expression of *RXRr* in heads of tadpoles exposed 21 days was suppressed by DBP and MBP. Opitz et al. [29] had evaluated the utility of mRNA expression of *TSHα* and *TSHβ* as a molecular endpoint to detect alteration in thyroid system function, which made up the methodological limitations in characterizing the thyroidal status of the differential treated tadpoles by means of analysis of serum T_4_, T_3_ and TSH concentrations. With regard to the mRNA levels of *TSHα*, no significant difference was observed between the high concentrations (10 and 15 mg/L) of DBP/MBP groups and the control group at exposure day 21. The mRNA level of *TSHα* increased significantly only in 2 mg/L DBP/MBP treated groups and 10 mg/L MBP treated group compared with the control group at stage 57. *TSHβ* is induced by the two compounds at 2 mg/L but only at exposure day 21. At 10 and 15 mg/L exposure levels, the mRNA level of *TSHβ* increased significantly only in 10 and 15 mg/L MBP treated groups compared with the control group at exposure day 21. However, no up regulation of *TSHβ* was observed for DBP or MBP at any concentration. The mRNA level of *TSHβ* decreased significantly only in 10 mg/L DBP treated group and 15 mg/L DBP/MBP treated groups at stage 57. We speculated that the capacity of the hypothalamus-pituitary-thyroid (HPT) axis to maintain sufficient TH levels may be exhausted by DBP/MBP in different manner. In addition, negative feedback of circulating TH and accumulating endogenous T_3_ may also have some impact on the regulation of genes expression in *X. laevis* treated with DBP/MBP. This speculation needs to be investigated in further studies. Our observation was consistent with previous study that mRNA expression of both TSH subunits was more remarkably increased at 25 mg/L ethylenethiourea (ETU) than at 50 mg/L ETU [29], which may be attributed to reduced functional properties of the median eminence, a structure provids the neurovascular link between neurosecretory centers in hypothalamic preoptic area and pituitary required thyroid hormone for proper function differentiation [35].

SMRT (co-repressor) is associated with the unliganded TR-RXR heterodimer bound TRE to suppress the transcription of TR response genes [36]. Moriyama et al. [37] revealed that BPA suppressed transcriptional activity by inhibiting T_3_ binding to the TR and by recruiting corepressor on the promoter. However, no previous report indicated the effects of DBP and MBP on recruiting co-repressors. We constructed the plasmids pVP16-L- hTRβ and pM-SMRT to investigate whether the two chemicals could recruit co-repressor SMRT. In the present study, we found that DBP and MBP enhanced the interactions between SMRT and TR in a dose-dependent manner even though the addition of 1 nM T_3_ released the interactions. Furthermore, MBP displayed stronger activity. Our result suggested that the two test chemicals could impair thyroid hormone action by suppressing its transcriptional activity. This is the first report that DBP and MBP can antagonize T_3_ action at transcriptional level. Moreover, previously we found that DBP and MBP also could display anti-thyroid hormone activity through TR by TR mediate luciferase assay [8].

EDCs interfere with thyroid hormone dependent gene regulation by disrupting the recruitment and function of co-repressors and/or co-repressors to target gene promoters. This in turn may also epigenetically alter the genome via DNA methylation and/or histone modification [38]. Therefore, we then pursued epigenetic analysis on *TRβ* gene. We specifically examined the methylation status of *TRβ* gene, because this epigenetic alteration is a common gene-silencing event in thyroid cancer in humans [39] and the DNA methylation patterns of *TRβ* gene were poorly characterized in *X. laevis* tadpoles treated with DBP and MBP. *TRβ* has been observed to play a prime role in inducing metamorphosis and shown to contain the TRE in its promoter region [40]–[42] and its induction in tadpoles tissues appears to be unique to thyroid hormone action, as a variety of other hormones which do not show a direct effect on this gene activity [43]–[45]. The level of *TRβ* mRNA increases in parallel with the elevation of endogenous TH by the thyroid gland, reaching a peak at the climax of metamorphosis, and then falling after metamorphosis [30]. Thus, *TRβ* is undoubtedly important for development and metamorphosis in *X. laevis*. In the present study, we used bisulfite sequencing to characterize the methylation patterns of *TRβ* gene promoter region in head tissue of *X. laevis*. Incompletely methylated clones were highly prevalent in head tissue of *X. laevis* treated with DBP and MBP, particularly MBP treated groups. *TRβ* gene has been characterized as a direct response gene to T_3_ and this gene contains several thyroid hormone response elements in its promoter region, thereby explaining the autoinduction by TH [34]. Abnormal DNA methylation may affect DNA-protein interactions and so may alter all processes in which such interactions occur. The promoter region of *TRβ* was analyzed for putative transcription factor binding site through AliBaba software (http://www.gene-regulation.com/pub/programs/alibaba2/index.htma) (data not shown) [46]. Since several transcription factors bound to the *TRβ* promoter region, aberrant methylated CpGs in the promoter region of *TRβ* may affect the binding of transcription factors, yielding clear information can result only from functional studies. We speculated that epigenetic alterations after exposure to MBP may underlie some of the effects on retardation of metamorphosis. The causal relationship of these epigenetic alterations remains to be elucidated.

In summary, our findings further argue for the urgent need to use *in vivo* animal models coupled with systematic molecular analysis to determine the developmental effects of endocrine disrupting chemicals. The current findings highlighted the danger of DBP and MBP as environmental thyroid disruptors. Therefore, DBP and MBP should be considered in risk assessments for human health.

## Supporting Information

Figure S1 Methylation status of promoter region of *TRβ* gene (18 CpGs) in head tissue of *X. laevis*. Each line represents an independent bisulphate-sequenced clone with the number on the right indicating the number of identical observations in the depicted sample (for each group a total of approximately 30 clones were sequenced). Open and closed circles indicate unmethylated and methylated CpGs, respectively. DBP: 2, 10, 15 mg/L (DBP 2, DBP 10, DBP 15). MBP: 2, 10, 15 mg/L (MBP 2, MBP 10, MBP 15). C: Number of clones. P: Pool codes with number of clones per methylation patterns.
(TIF)

Table S1 Sequences of primers for qPCR and bisulfite-PCR analyses.
(DOC)
