# Supplementary figures and images for: Thyroid Disruption by Di-n-Butyl Phthalate (DBP) and Mono-n-Butyl Phthalate (MBP) in Xenopus laevis
Source: PLoS One. 2011 Apr 22;6(4):e19159. doi: 10.1371/journal.pone.0019159 (PMC3081329; doi:10.1371/journal.pone.0019159)

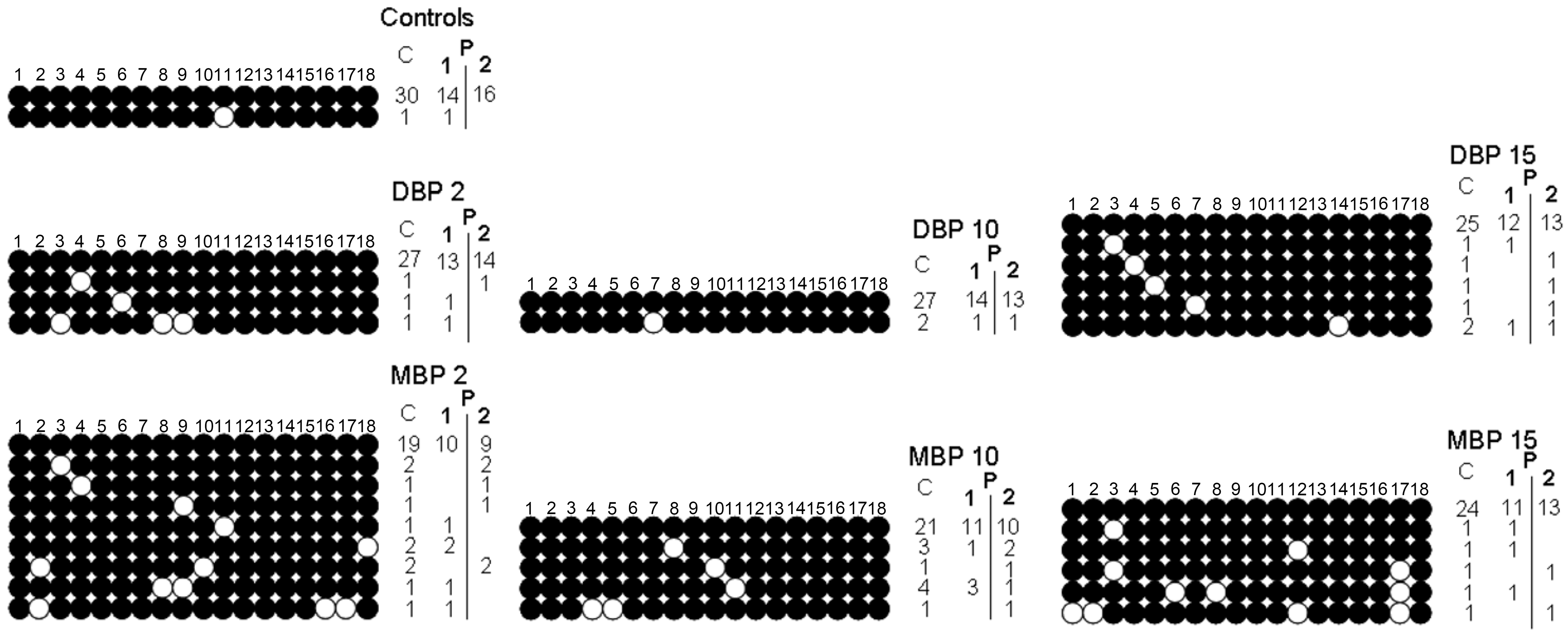

Supplement: Figure S1 — Methylation status of promoter region of TRβ gene (18 CpGs) in head tissue of X. laevis. Each line represents an independent bisulphate-sequenced clone with the number on the right indicating the number of identical observations in the depicted sample (for each group a total of approximately 30 clones were sequenced). Open and closed circles indicate unmethylated and methylated CpGs, respectively. DBP: 2, 10, 15 mg/L (DBP 2, DBP 10, DBP 15). MBP: 2, 10, 15 mg/L (MBP 2, MBP 10, MBP 15). C: Number of clones. P: Pool codes with number of clones per methylation patterns. (TIF) [file pone.0019159.s001.tif]
