# Supplementary material for: Thyroid Disruption by Di-n-Butyl Phthalate (DBP) and Mono-n-Butyl Phthalate (MBP) in Xenopus laevis
Source: PLoS One. 2011 Apr 22;6(4):e19159. doi: 10.1371/journal.pone.0019159 (PMC3081329; doi:10.1371/journal.pone.0019159)
Supplement: Table S1 — Sequences of primers for qPCR and bisulfite-PCR analyses. (DOC) [file pone.0019159.s002.doc]

Table S1. Sequences of primers for qPCR and bisulfite-PCR analyses.

| **Gene** | **Direction** | **Primer sequence (5’-3’)** | **References** |
| --- | --- | --- | --- |
| **qPCR Primers** | | | |
| TRβ | Forward | TGTTCAGAAACCTGAACCCACACAA | (30) |
|  | Reverse | TCCACCCTCGGGCGCATTAA |  |
| RXRγ | Forward | AGAAGCCGAGAGAAAAGTGATACA | (30) |
|  | Reverse | TGGATCCACTGCCAGCTCTGCTT |  |
| TSHα | Forward | ACGGGTCACAAGTGATGG | (29) |
|  | Reverse | GGGATCACATCATGCAGATGA |  |
| TSHβ | Forward | AGAGTGCGCTTACTGCCTTG | (29) |
|  | Reverse | GGTAGGAAAAGAGCGGGTTC |  |
| gapdh | Forward | CTCATGACAACAGTCCATGCTTTC |  |
|  | Reverse | CTCTGCCATCTCTCCACAGCTT |  |
| **Bisulfite Analysis Primers** | | | |
| TRβ | Forward | GTTTTTAGTTTAAGGTTTTTTGG |  |
|  | Reverse | AATAATATACAAATTAATATTTCCCC |  |
